# Beyond multiculturalism: revisioning a model of pandemic anti-racism education in post-Covid-19 Canada

**DOI:** 10.1186/s41257-021-00060-7

**Published:** 2022-01-28

**Authors:** Ling Lei, Shibao Guo

**Affiliations:** Werklund School of Education, University of Calgary, 2500 University Dr NW, Calgary, AB T2N 1N4 Canada

**Keywords:** Multiculturalism, Racism, Covid-19, Pandemic anti-racism education, Critical race theory

## Abstract

Canada was the first country in the world to establish multiculturalism as its official policy for the governance of diversity. Canadian multiculturalism has gained much popularity in political and public discourses in the past 50 years, and it has also received no less criticism as to its effectiveness in addressing issues of racism. There have also been ambiguities over the meaning and intention of multiculturalism, leading to divergent understandings of multiculturalism as an ideal of inclusion and equity, on the one hand, and a mere political rhetoric, on the other. On the occasion of celebrating the 50^th^ anniversary of Canada’s official multiculturalism policy, this article re-visits Canada’s multiculturalism by reviewing its history and ethos and critically examining its actual effects as manifested during the Covid-19 pandemic in Canada. The rise of anti-Asian racism, anti-Black racism, and anti-Indigenous racism incidents in the pandemic reveals that multiculturalism has in effect, sustained a racist and unequal society of Canada with racism entrenched in its history and ingrained in every aspect of its social structure. Multiculturalism tolerates cultural difference but does not challenge an unjust society premised on white supremacy. The anti-racism movement mobilized by racialized communities in Canada indicates that multiculturalism has failed to respond to racialized communities’ pressing demand for social change and action for social justice. The article concludes with a proposed alternative framework to multiculturalism, that is, pandemic anti-racism education model, to centre the issue of race and racism in an action-oriented, inclusive, and empowering approach toward a future of a just society.

## Introduction

The year 2021 marks the 50^th^ anniversary of the official multiculturalism policy in Canada. Fifty years ago, Canada was the first country in the world to establish multiculturalism as its official policy for the governance of diversity in 1971. The past 50 years have witnessed its appeal and popularity in political and public discourses, and in terms of the amount of government and civil organization programs. Its success can be reflected by the international acclaim Canada has received through enacting a governance model for other immigration countries, by the positive boost to Canada’s ambitious immigration levels plan, as well as by the fostering of a collective diversity-tolerant mindset with Canadians (Fleras 2018). However, despite its perceived success and strength as opposed to the outright Eurocentric and racist policy in its preceding period, its ability to withstand the challenges of the current times after 50 years of implementation remains unclear. In retrospect, there has never been a lack of critique and controversies over the meaning of Canadian multiculturalism and its actual effects over the course of its existence. Meanwhile, the new realities of massive transnational migration, and social fluidity and changes have led to the questioning of multiculturalism’s continued legitimacy (Grzymala-Kazlowska and Phillimore 2018). Particularly, the many incidents of racial discrimination triggered by the Covid-19 pandemic have further exposed the historical process of racialization and deeply entrenched racial ideology masked by Canada’s official framework of multiculturalism (Guo and Guo 2021). It seems clear that Canada’s multiculturalism policy is now at a critical crossroad if it is committed toward social equality and inclusion (Fleras 2019).

On the occasion of celebrating the 50^th^ anniversary of Canada’s official multiculturalism policy, it is necessary to revisit multiculturalism at the crossroad of pandemic racism during Covid-19 in Canada. It seems there is an urgent need to engage in critical deep reflections on the rhetoric of Canadian exceptionalism presenting the country as a culturally diverse and inclusive nation. The article is organized into five parts. It first presents an overview of the history and ethos of the multiculturalism policy. Next, it discusses the research methodology which draws on critical discourse analysis. Then, it critically reviews challenges to multiculturalism as they are manifested during Covid-19, including anti-Asian racism, anti-Black racism, and anti-Indigenous racism. The fourth section illustrates anti-racism movement across Canada as grassroot actions to fight against racism. The paper concludes with a suggested framework that centres on pandemic anti-racism in taking us beyond multiculturalism.

## Multiculturalism turns 50: the history and ethos of Canadian multiculturalism

The adoption of multiculturalism policy reflects the realities and changes to Canada’s immigration history (Meister 2021). Initially, multiculturalism has just been a description of the social demographic fact in Canada since the confederation of the country in 1867, when there were three founding ethnic groups, Indigenous peoples, French, and British. The latter two colonized the land of the Indigenous peoples and claimed power as the two dominant ethnic groups (Wong and Guo 2015). After confederation, there have always been immigrants to Canada from various countries, but mainly because of the legislation of racist Immigration Act of 1896 and 1952, which promoted a White Canada nation building agenda, racialized immigrants were prohibited or restricted from immigrating to Canada. Other systemic barriers targeting immigrants of colour also added to the exclusion of ethnic groups that the dominant White Canadians considered undesirable for “a white man’s country”. These barriers include legislations such as the Chinese Immigration Act (1885) that imposed a head tax on Chinese immigrants, the Chinese Exclusion Act (1923) that banned all Chinese immigrants until its repeal in 1947, the denial of entry of SS Komagata Maru ship with Indian refugees fleeing from the First World War, the internment of Japanese immigrants during the Second World War, the ban on immigration of Blacks in 1911, and the many discriminatory practices that subjugate racialized minorities to the margins (Anthony 2020; Guo and Wong 2018). Meanwhile, the Indigenous people have not only been deprived of their lands, but their rights have long been disregarded, and their existence as a founding group in Canada has also been diminished and denied for a long time.

This scenario of overt racialization and colonization has not undergone much change until the post-WWII period. The increased international pressure on countries to abandon overtly racist immigration policies and a post-war economic boom in Canada have precipitated the conception of a new governance and immigration model. Since then, the formation and development of multiculturalism as a public policy has evolved in three stages: 1) incipient stage (pre-1971); 2) formative stage (1971-1981); 3) institutionalization stage (1982-present) (Dewing 2013). The conception of multiculturalism policy in 1971 was spurred by a national unity crisis in the incipient stage, where the Quebecois raged its secessionist movements in the 1960s. The federal government then implemented a series of reforms to enhance the status of French Canadians, affirming English and French bilingualism and biculturalism to strengthen the equality of British and French as the “founding nations” of Canada (Kymlicka 2015). However, these “duality” initiatives caused concern and objection from other ethnic groups in Canada, particularly the other white ethnic groups from Europe such as the Ukrainians, Italians, and Poles, as they fear that the emphasis on unity between the British and the French would be achieved at their expense. These ethnic groups then demanded official recognition of the ethnic diversity as well as financial support for ethnic groups in Canada to maintain their cultural identities.

Such multicultural movement in the 1960s influenced the recommendations made by the Royal Commission on Bilingualism and Biculturalism (RCBB) appointed by the Pearson government in 1963. The recommendations of the Commission were responded to by Prime Minister Pierre Elliott Trudeau, who proclaimed in the House of Commons on October 8, 1971, that his government policy is “multiculturalism within a bilingual framework” (Blanding 2021). The announcement marked the beginning of the formative stage of multiculturalism. For over ten years since then, the federal government allocated funding dedicated to the implementation of programs that foster ethnic culture development, language teaching and learning, and cultural exchanges (Dewing 2013). Multiculturalism in the formative stage is believed to be focused on celebrating cultural differences and objectified cultures such as dress, dance, and food (Fleras and Kunz 2001).

The institutionalization stage (1982-present) is characterized by formal institutionalization of the multiculturalism policy in legislation. In 1988, the Canadian Multiculturalism Act was enacted, which endorsed multiculturalism as a central feature of Canadian citizenship and upheld every Canadian’s freedom to his or her cultural heritage. Other institutionalization efforts include the recognition of Canada’s multicultural heritage and the inclusion of one’s ethnic origin and race in the Canadian Charter of Rights and Freedoms (1982), the establishment of the Canadian Race Relations Foundation Act (1991), the Employment Equity Act (1986, 1998) to eliminate discrimination against under-represented groups in the workplace, including Indigenous people, visible minorities, people with disabilities, and women. Other more recent institutionalization efforts include the establishment of Canadian Multiculturalism Day (June 27) in 2002, Asian Heritage Month (May) in 2002, Black History Month (February) in 2008, and Canada’s Action Plan Against Racism in 2005 as well as its relevant strategies. In this stage, the foci of Canadian multiculturalism evolved to one of “equity” in the 1980s, “civic participation” in the 1990s, and “integration” in the 2000s (Fleras and Kunz 2001; Kunz and Sykes 2007).

In terms of recognition of ethnic groups’ cultures, the conception of multiculturalism is indeed a radical departure from an assimilationist and overtly racist approach in the past, offering acknowledgement of the ethnic diversity in Canada. Yet, its ultimate aim was at defending the political power of the British and French groups and the biculturalist narrative of Canada’s nationhood. It is believed that Trudeau’s vision of a multicultural Canada is of “a French-English bilingual, but not binational country, coupled with individual rights and freedoms – something he called the ‘Just Society’” (Blanding 2021, p. 18). However, with the British and French being in privilege and power as a precondition for a “Just Society”, the extent to which other ethnic groups can exercise individual rights and freedoms seems to be quite circumscribed. Meanwhile, it must be noted that multiculturalism policy was not created with non-European immigrants in mind but rather was “demanded by, and designed for, European immigrant groups” (Kymlicka 2008, cited in Meister 2021, p. 13). In other words, those racialized non-white ethnic groups, such as people of African, Asian, and Indigenous descent, were not regarded as stakeholders in the original conception of multiculturalism (Meister 2021). Besides, multiculturalism was depicted as an imagery of a “mosaic” (Graham 1998; Porter 1965), which perfectly masked the power relations in Canada (Warren 2021).

The three stages of multiculturalism reflect a change of emphasis and focus from culture to race relations and social justice (Ghosh 2011). All three dimensions are important and positive promises of multiculturalism. Kymlicka (1995) claimed that heritage culture constitutes a critical aspect of an individual’s identity. Thus, the recognition of minority groups’ rights and freedom to cultural differences can facilitate integration and is compatible with the ideology of a liberal state. In the same vein, Taylor (1994) asserted that an individual’s membership in a community is vital to his or her identity and thus one’s community should receive due recognition both politically and socially. At the same time, Taylor argues, people should make efforts to achieve cross-cultural understanding through dialogue. From their perspectives, multiculturalism in Canada is conceived as cultural pluralism with limited attention to power relations. Scholars like Fleras (2019) illuminated the limits of a model of multiculturalism in Canada as cultural pluralism, as ethnic groups’ right to inclusion is accepted on the condition that it does not challenge the hegemonic racial hierarchy imposed by the dominant social group. Despite a critique of multiculturalism as a state-centric governance regime, an alternative solution is believed to be worked out through a more interactive pluralism approach, or through the agency of individuals in intercultural interactions (Wong 2015). However, as critical multiculturalism scholars have pointed out, multiculturalism that is conceived as issues for the visible minority “other” to deal with cannot ensure social equality and just race relations in Canada. Rather, the need is to involve both the dominant and minority groups to become equally aware of unequal power relations, the impact of difference on social positions, the marginalizing experiences of those who are deemed “different” other, and to transform unjust institutional practices (Ghosh 2011). As critical multiculturalism literature suggested, multiculturalism will only reproduce ethnic marginalization and stratification when the society remains a systemically unjust and unequal operation (Wong 2015).

It seems that multiculturalism tends to face increased criticism and challenge to its legitimacy in two situations. On the one hand, critical literature on multiculturalism has shown that a rise in strong opposition to multiculturalism occurs when there are increasing conflicts in global politics, increasing immigration and ethnic diversity, and increasing globalization. As Wong (2015) observed, there was a huge increase in academic and public discourse in the retreat of multiculturalism since the 1980s and particularly in the 1990s and the post-9/11 event period. The increase in the visible minority population from non-European countries in the 1980s lent to propositions that multiculturalism’s emphasis on cultural difference and cultural relativism will cause social fragmentation (Bibby 1990). Similarly, in a context of political conflict between the Islamic world and the West, as manifested in the re-emergence of racism against the Muslim population in France over headscarf issue in the 1990s, multiculturalism was believed to bring about global and domestic clashes of cultures (Huntington 1993; Silverman 1992; Wieviorka 1998). The 9/11 event further fostered angst against multiculturalism as it was claimed that multiculturalism failed to achieve minority integration or address social inequality. Instead, it produces ethnic segregation and reproduces ethnic stratification (Abbas 2005). On the other hand, the changing face of cultural diversity in Canada over the last five decades together with immigrants’ transnational patterns of integration has led to the argument that multiculturalism is dated for managing diversity in a hyper-diverse, “post-multicultural” world (Fleras 2015; Vertovec 2010; Wieviorka 2014). Indeed, Canada’s demographic has undergone significant changes since the implementation of the multiculturalism policy in 1971. On the one hand, the percentage of immigrants from non-European and non-U.S. regions rose from about 14% in 1965 to almost 70% by 1985. The visible minority population doubled from a negligible 5% at the beginning of the 1980s to 10% in 1991. Accordingly, in Canada’s metropolitan areas such as Toronto and Vancouver, the percentage of visible minorities also doubled, reaching 26 and 24% respectively in 1991. In 2001, Canada’s visible minority population increased to 13.4%. Ten years later in 2011, almost one fifth of Canada’s population were visible minorities. The 2016 census has revealed a 3.2% increase in the five-year period since 2011, with a total of 7,674,580 people, or 22.3% of the population as visible minorities. In large metropolitan areas such as Toronto, Vancouver, and Calgary, the proportions were 47, 45.2, and 28.1%, respectively. The variety of ethnic origins has also grown at the same time and there were over 250 ethnic origins listed in the 2016 census. On the other hand, the percentage of the two dominant groups, British and French, fell from 88% in 1901 to 63% in 2001 and further down to 46.1% in 2016 (Statistics Canada 2008, 2013, 2017). Such growing ethno-cultural diversity in the Canadian population means that multiculturalism policy needs to accommodate and reflect the needs and rights of a much wider ethnic diversity and to recognize the changing fact that with increasing visible minority immigrants settling in large cities like Toronto and Vancouver, the visible minorities will soon become the majority in these urban areas (Wiseman 2018; Yu 2018).

## Research methodology

This article adopts critical discourse analysis (CDA) as its research methodology underpinned by the interpretive and critical research paradigms to inform data collection and analysis (Wodak and Meyer 2009). It focuses on social issues of inequality with an aim to expose unequal power relations and hegemonic knowledge, to make sense of the formation of such power difference and hegemony through critical analysis of various kinds of discourses, and to bring about change through critical understanding (Van Dijk 2007). Analysis is achieved through descriptive and explanatory procedures in which the values, beliefs, and ideologies behind various discourses, or texts, are identified and critically evaluated within their socio-historical contexts. It can include both a focus on structure and a focus on action (Fairclough 2001). Following a Foucauldian perspective, discourse is formed through knowledge and practice solidified and normalized through history. Discourse is imbued with power as powerful actors which influence and impose hegemonic beliefs, practices, and structures (Foucault 1970, 1988). CDA examines the historical roots of beliefs, practices, and structures to emancipate those dominated by hegemonic powers. In this article, news reports in popular media are used and described as texts. Themes in these texts are identified and then critically analyzed through discussion with another source of text, that is, scholarly publications, to examine the external social relations and structures shaping the formation of the texts. Alternative discourses for moving toward a more just society for the racialized community in Canada are then developed.

Data for this paper were derived from online media reports search in Google.ca. With foci on both structure and agency, key terms used for the search consisted of two strands, including those relevant for racism and those relevant for action against racism. Thus, key terms used included “Asian Canadian” “Black Canadian”, “Indigenous”, together with “racism” and “Covid-19”. Further search was conducted with key terms such as “social action”, “activism”, “social movement”, “advocacy”, “leadership”, and “initiative”, together with “racism” and “Covid-19”. The search was delimited to news reports in 2020 and 2021 and primarily to mainstream news media in Canada. Meanwhile, scholarly publications included for discussion in this article are primarily academic articles and research reports pertaining to the theme of racism against the racialized community in Canada conducted in the last ten years.

## Multiculturalism at a crossroad: the rise of racism during Covid-19

Five decades since its implementation, Canada’s multiculturalism has arrived at a crossroad where multiculturalism’s ideal of inclusion, equality, and social justice could be obstructed by discourses of the retreat and failure of multiculturalism, leading to its complete abandonment. Particularly, increased incidents of anti-racism during the current Covid-19 pandemic in Canada are reminiscent of racist incidents and ethnic tensions in history that gave rise to doubts of the usefulness and effectiveness of multiculturalism. Meanwhile, elevated voices of protest from the ethnic minority community against racist behaviours during Covid-19 have also indicated that it is a pressing moment to revisit multiculturalism in Canada: What ideals of multiculturalism do we uphold and how are we going to proceed in practice to work toward the realization of such ideals? This section discusses current challenges to multiculturalism by critically analyzing scholarly publications and incidents that were reported in popular press during the pandemic pertaining to racism against Asians, Blacks, and the Indigenous people^1^ during Covid-19 in Canada. Asians, Blacks, and the Indigenous people are referred to as the racialized community, a term acknowledging the common experiences of discrimination and marginalization faced by non-Caucasian and non-white people in Canada.

### Anti-Asian racism

Since the outbreak of COVID-19, there has been a surge in racism and xenophobia across Canada towards Asian Canadians, particularly those of Chinese descent (Guo and Guo 2021). They have been spat on, verbally abused, and physically attacked. Many became the scapegoat who were wrongly blamed for spreading the virus just because it was first reported in China. They are shouted to “go home” although some of them are born in Canada who have never visited their ancestral lands. Many of them have also become the target of hate crimes. Since the outbreak of the global pandemic, there has been a significant rise of reported hate crimes perpetrated against Asian Canadians resulting primarily from ignorance, fear and misinformation. In Vancouver, for example, hate crime incidents targeting Asian communities rose by 717% in 2020 compared to 2019, the highest per Asian capita in North America (Liu 2021). Some of its members are stigmatized because of the virus and their properties are vandalized. They have been depicted as “weak, sickly, diseased, and foreign, and therefore ‘undesirable’ citizens” (Guo and Guo 2021, p. 204). As victims of racial discrimination, Asian Canadians have subsequently experienced high levels of anxiety, trauma, and desperation.

One recent news report by Canadian Television Network (commonly known as CTV) (2021) revealed, by citing findings of several Asian advocacy groups, that after one year into the pandemic, there has been more than 1,000 cases of racist attacks against Asians in Canada. The cases were reported through online platforms such as covidracism.ca and elimin8hate.org. There are more than ten types of racist incidents reported, with the top type being verbal harassment, followed by physical aggression or unwanted physical contact, being coughed at or spat on, and vandalism (The Chinese Canadian National Council [CCNC] 2021). According to this report, most of the cases were reported from Ontario and British Columbia (40 and 44% respectively). Almost 60% of case victims were women and there were also a high proportion of physical assault victims as children, adolescents, youth, older adults, and seniors. An earlier survey of more than 500 Chinese Canadians revealed that respondents were exposed to racist messaging on social media and were made to feel as if they posed a health and safety threat to others (Angus Reid Institute 2020).

A prominent characteristic of the experiences of racism for Asian Canadians is intersection of racism and other identity markers such as gender and age. Research by the Learning Network at the Centre for Research and Education on Violence Against Women and Children (VAW Learning Network) (2021) pointed out that Asian women are particularly susceptible to racism and discrimination during the Covid-19 pandemic because of xenophobia of immigrants as “perpetual foreigners” and gender-based violence that have existed both historically and in contemporary Canada. Historical discrimination against Asian women for building a “white Canada” and contemporary immigration policies and workplace practices that exclude and subordinate Asian women to the bottom of racial and social class hierarchies have shaped the negative portrayal and stigmatization of Asian women as inferior, thus threatening their safety and wellbeing particularly in times of ethnic tensions.

A study on the experiences of and impacts of Covid-19 on older Chinese immigrants in Canada indicated that the racialized community in Canada is disproportionally affected by Covid-19 as they faced higher physical health and psycho-social risk than their Canadian-born counterparts. Chinese senior immigrants in the study felt that there has always been discrimination and racism, but they have become more explicit during the pandemic. The feeling of being blamed as carriers of the virus has created discomfort, distress, and the inclination of further isolation from others (Wang et al. 2021).

### Anti-Black racism

Anti-Black racism in Canada during Covid-19 was initially triggered by the death of a 29-year-old black Toronto resident Regis Korchinski-Paquet. On May 27, 2020, Regis fell 24 storeys from a balcony of the apartment where her family lived. At the time of the incident, Regis was with the police alone in the apartment and the police refused to reveal what happened right before her death. Regis’s family raised concerns that Regis’s death may be related to racism against the black. Thereafter, news report on this case immediately ignited anger from the public and protests against anti-black racism and police brutality soon followed (Canadian Broadcasting Corporation [CBC] News 2020). These protests were joined by those in solidarity with lives lost to racism in the U.S. at that time, with the most appalling case being the murder of George Floyd by a Minneapolis police officer. This case became the breaking point of the most current wave of civil rights movement in the U.S., referred to as “Black Lives Matter” (BLM) movement. The BLM movement in the U.S. together with the Regis case in Canada have brought to the spotlight the issue of police brutality against the black in Canada. As CBC News’ research on fatalities at the hands of the police shows, Black and Indigenous people are over-represented in death caused by police violence (Singh 2020).

In addition to the 2020 BLM movement, the health and social risks suffered by Black Canadians during Covid-19 also exposed the long-standing issue of racism against the Black community. A report from the African Canadian Civic Engagement Council and Innovative Research Group (2020) demonstrated that Black Canadians are more vulnerable to the negative physical, socioeconomic, and psychosocial impacts of Covid-19. They are more likely to report Covid-19 symptoms as they are more likely to have jobs that require face-to-face interaction and to commute to work via public transit. Moreover, Black Canadians are reported to be more likely to experience layoff or reduced working hours, and thus, more worried about paying rent and household finances in general. In addition, refusal to collect and publish race-based health data on Covid-19 in Canada was an indicator of ignorance and trivialization of health inequities for the racialized community, including Black Canadians (Bowden 2020; Osman 2021).

The BLM movement and the disproportionally high vulnerability of Blacks, particularly during Covid-19, should be understood within the historical context of structural racism, colonialism, and global capitalism (Kihika 2020). As Kihika pointed out, as a multicultural country Canada always claims to be “better” than its U.S. neighbour, which seems to be caught by a much more severe issue of racism and to hold a more overt attitude of hostility toward the racialized community, particularly the Blacks. Canada also seems to have a tradition of ignorance about racism. Thus, the issue of racism is believed to be “over there” (in the U.S.) and “in the past”. However, the reality of the vulnerability of Black Canadians during Covid-19 shows that Canada’s anti-Black racism may seem subtle, but its impact is felt and experienced substantively by Black Canadians, who are subject to poverty, inequity, and insecurity due to racialization (Block and Galabuzi 2018; Dei and Lewis 2020). Meanwhile, institutionalized structures of white supremacy in Canada that marginalize racialized communities, including Black Canadians, have never been erased. What is being erased, hidden, or silenced from the public is the recognition of Black Canadians’ experiences of marginalization and social injustice as something existing “here”, “now”, and “always” in Canada and something rooted in and sustained by Canada’s institutional structure (DasGupta et al. 2020).

The strong association between police brutality against the Blacks in Canada, higher rates of Covid-19 among Black Canadians and the intersection of their low socio-economic status, low level of education, lower-paying employment with high risk of exposure to Covid-19, and being a visible minority is a galling reminder of the enduring impact and insidious manifestation of historical racism against Blacks. As Mianda (2020) argued, Black deaths in police brutality and deaths due to Covid-19 appear to be separate concerns, but they are in fact “linked expressions of deeply entrenched anti-Black racisms” (p. 3).

### Anti-Indigenous racism

The most shocking news during Covid-19 that awakened Canada to its history of colonization and racism against the Indigenous people was the discovery of more than 1,300 unmarked graves near former residential schools. These graves are believed to contain remains of mostly Indigenous children who were separated from their families and forced to live in residential schools (The Globe and Mail 2021). More than 150,000 Indigenous children between the age of 4 and 16 were sent to more than 139 residential schools between the 1870s and the 1990s until the last residential school closed in 1996. The unmarked graves remind people of the sufferings of Indigenous children, who experienced physical and mental abuse, malnutrition, lack of medical care, and sexual assault, to name just a few, leading to more than 4,100 deaths of children in the schools (The Globe and Mail 2021; Union of Ontario Indians 2013). Three unmarked graves were found and reported in summer 2021. There are 751 such graves found in Cowessess First Nation in Saskatchewan near the former Marieval Indian Residential School, 215 graves found in the former Kamloops Indian Residential School in B.C., and 182 unmarked graves found in the former St. Eugene’s Mission School near Cranbrook, British Columbia.

While state-sanctioned violence and injustice against Indigenous people seem to be tucked in the past, systemic racism against Indigenous people is still prevalent and all-encompassing in Canada. The experiences of Indigenous people during Covid-19 have brought to light the impact of everyday experiences of inequity in times of emergency. According to Hawthorn (2021), the Indigenous community is disproportionately hurt by the pandemic with a much higher Covid-19 case rate than the Canadian average due to poor living and health conditions including food scarcity, overcrowded housing, contaminated water, and substandard health care. As explicated by a report by Public Health Agency of Canada (2021) on the State of Public Health in Canada during Covid-19, the implementation of public health behaviours such as frequent hand washing, physical distancing, and self-isolation is a challenge in Indigenous communities where clean water may not be readily available and space for isolating infected people may not be available with a lack of housing. In terms of mental health, government policies on lockdown triggered Indigenous people’s traumatic memories of colonization in the past when white colonizers dismissed the rights and freedoms of Indigenous people. Many Indigenous people felt reluctant to approach health care professionals and uncomfortable accessing healthcare service during the pandemic because of past experiences of systemic racism in healthcare.

While the above report is concerned primarily about Indigenous people in remote and rural areas, a policy and practice review article commented that the experiences of Indigenous people living in urban areas are also worthy of attention, as between 65% and 80% of the two million Indigenous people in Canada live in urban communities (Howard-Bobiwash et al. 2021). The issue of systemic racism against Indigenous people in urban areas was made invisible by the Canadian government as no disaggregated data on Indigenous peoples’ health status is recorded or published. Besides, while there are Indigenous representative organizations ready and available to engage with in the urban areas, they are not consulted in terms of Covid-19 intervention. Thus, the erasure of Indigenous people’s health status and the neglect of Indigenous people’s treaty rights, including the right to self-governance during the pandemic, manifests Canada’s pandemic colonialism, which sustains practices of settler colonialism with mere rhetoric of structural oppression and Indigenous rights (Howard-Bobiwash et al. 2021). Racism against Indigenous people during the pandemic is also exposed in the tragic death of an Indigenous women in Quebec. An Atikamekw woman, Joyce Echaquan, a mother of seven, died on September 28, 2020, because of mistreatment she received in a hospital north of Montreal. The hospital staff believed Echaquan was suffering from withdrawal, so she was restrained and left alone in her hospital room. However, there was no evidence of this. Hospital staff infantilized Echaquan and labelled her as a manipulative drug abuser because of their prejudice and bias against Indigenous people. They also hurled racist remarks at Echaquan. Despite these findings, the Premier of Quebec, François Legault, refused to acknowledge the existence of systemic racism, which he believed to be instead, an issue for some individuals and an issue that only existed in the past when there were residential schools (Nerestant 2021).

As research shows, such discriminatory healthcare treatment of Indigenous people has been a recurring problem rather than occasional incidents occurring only during the current pandemic (Skosireva et al. 2014; Wylie and McConkey 2019). As Mawani (2020) pointed out, the destruction of Indigenous people’s health is part of the settler colonial project of Indigenous disappearance, which has happened for over hundreds of years and is still ongoing today. In addition, the higher vulnerability of Indigenous people to health challenges cannot be viewed as individual issues as colonization and racism are conditions that created the vulnerability in the first place. These conditions create a race-based hierarchy of the value of lives, where the lives of Indigenous people and other racialized people are devalued and destroyed. Meanwhile, as Perry (2021) argued, there has been an invisibility of “whiteness” in critical race scholarship. Such invisibility tends to naturalize colonization and dispossession, making the history of colonization and racialization appear “inevitable and benign”. Without a direct confrontation with whiteness, power and domination that have been ascribed to whiteness remain intact, on the one hand, and the suffering and discrimination experienced by Indigenous people remain, on the other hand, unrecognized, obscured, and unaddressed. Moreover, although most of the extant literature on racism against Indigenous people tend to focus on Indigenous health issues, anti-Indigenous racism is perceived and experienced by Indigenous people in all social institutions and at multiple levels, including individual, collective, institutional, and cultural levels (Benoit et al. 2019).

In addition, Indigenous people in Canada also suffered race-based police brutality and this issue was thrust into spotlight during Covid-19 following the mass protests against police brutality targeting Black communities in the United States. A string of police violence incidents against Indigenous people were reported from March to June 2020. For example, a 26-year-old First Nations mother, Chantel Moore, was shot dead by the police in New Brunswick after the police was called to her apartment for a wellness check. During the same week, a Royal Canadian Mounted Police (RCMP) officer in Nunavut hit a 22-year-old Inuk man with the door of a moving truck. One week later, Rodney Levi was shot and killed by an RCMP officer during a barbeque gathering outside a church pastor’s residence in News Brunswick. In another incident, Athabasca Chipewyan First Nations Chief Allan Adam was punched, tackled, and choked by RCMP in Alberta during his arrest over an expired license plate. It was reported that in this period alone, six Indigenous people have died in police violence in Canada (Britneff 2020; Cecco 2020; Graham 2020).

Despite RCMP and many politicians’ reluctance to admit the existence of systemic racism behind police brutality targeting the Indigenous community in Canada, quantitative crime-related data have shown that the Indigenous community is indeed more likely than white Canadians to become victims of police force. There have been higher rates of police-reported crimes in Indigenous communities and an overrepresentation of Indigenous people in prison population, which indicates discriminatory policing and discriminatory stereotype of Indigenous people (The Council of Canadian Academies [CCA] 2019; Stelkia 2020). While such discrimination can be traced back to the history of colonization of Indigenous people, the denial of the presence of systemic racism against Indigenous people in the Canadian criminal justice system today allows for the normalization of such discriminatory practices, the perpetuation of negative stereotype, and the ignorance of failure to counter this issue (Cao 2014).

## Calling to action: anti-racism movement in Canada

It can be seen from the analysis of media reports and scholarly publications that critically discuss anti-racism incidents during the pandemic, Canadian multiculturalism policy is at a crossroad. Fifty years after its implementation, its legitimacy is at stake. As a federal policy, it runs the risk of being too distanced from people’s everyday practices, and thus being rendered a mere rhetoric. In addition, it runs the risk of failing to effectively meet the pressing demands of racialized communities for equity and social justice. As the visible minority population expands and its visibility grows through various cultural organizations and ethnic organizations (Guo and Guo 2011), racialized communities today demand not only recognition of their heritage culture, or more conversations about diversity, but also a heightened awareness and better social mobilization capability to demand immediate action and change for equity and social justice. This is clear through the scale of anti-racism protests, advocacy, and social mobilization occurred during the pandemic. For instance, Chinese Canadians across the country organized numerous anti-racism campaigns and educational events using online meeting platforms such as Zoom and social media platforms such as WeChat in response to discrimination against Asian immigrants during Covid-19. The Chinese Canadian National Council for Social Justice (CCNC-SJ) organized a series of initiatives to raise public awareness about anti-Asian racism and call for proactive actions to fight against racism (Boisvert 2020; Patton 2020). The Action! Chinese Canadians Together (ACCT) Foundation (https://acctfoundation.ca/) and ACT2endracism (https://act2endracism.ca/) have been providing an online avenue for reporting racist incidents and for providing strategies and recommendations to educate people as to how to respond to racism and discrimination. Individuals, such as spoken poet Chris Tse, has called for anti-racism and highlighted Asian Canadians’ contributions to Canada via the online video platform of Youtube (CBC News 2021), and collective initiatives were made by Chinese immigrants such as online rally, online petition, as well as in-person rallies (Babych 2021; Chen 2020; We Canadian 2020).

The Black Canadian community has also been initiating acts of resistance against racism. In addition to the Black Lives Matter Movement and anti-Black racism protests that took place from coast to coast, many Black Canadian leaders and activists have started advocacy and long-term capacity-building initiatives. For example, Alliance for Healthier Communities (2020) has led rallies calling for the collection of race-based data on the impact of Covid-19 and has also issued a public statement by Black health leaders on Covid-19’s impact on Black communities in Ontario. Black Canadian business leaders have launched the Canadian Council of Business Leaders Against Anti-Black Systemic Racism and the BlackNorth Initiative to increase the representation of Black leaders in corporate Canada and to address multi-layered challenges facing Black Canadians (McNutt 2021). An immigrant and refugee support organization, Skills for Change, has launched the Black Leadership Institute on Social Action for Change to foster systemic change in addressing anti-Black racism (Skills for Change 2021).

Meanwhile, Indigenous people have continued to raise their voices for social justice and for truth and reconciliation following over a century of political organization and activism (Dyck and Sadik 2020). After the discovery of unmarked graves of Indigenous children died in residential schools, Indigenous advocates led marches, staged protests, and initiated awareness-raising public gatherings (French 2021). Indigenous people led searches of residential school sites and filed class-action lawsuits in seeking justice for harms they suffered in the past and for holding the federal government accountable for breaching charter rights. (Gilmore 2021; Stefanovich and Raycraft 2021). Indigenous leaders have initiated the Orange Shirt Day movement to raise awareness about the history and legacies of the residential school system in Canada and this grassroots commemorative day now takes place with the National Day for Truth and Reconciliation in Canada on September 30. The Indigenous-led social movement, Idle No More (INM), initiated in 2012, has continued to form alliance with the Black community and other marginalized Canadians to hold the Canadian government accountable for defending Indigenous people’s human rights and for addressing systemic racism and violence. Even during the Covid-19 pandemic, INM activists and their allies have organized many peaceful protests, including those against the federal government’s discriminatory Covid-19 assistance toward Indigenous people, and those against police brutality and racism against Indigenous people (Godin 2020). INM’s “Cancel Canada Day” protests after the discoveries of unmarked graves of Indigenous children at residential schools have successfully gained support from 80 municipalities in 10 provinces and territories, which decided to cancel Canada Day events to recognize the ongoing colonization genocide, oppression, and violation of human rights (INM 2021).

The struggles and acts of resistance of racialized communities against racism in Canada, as amplified in the current context of the pandemic, urge us to shift the gaze of multiculturalism from “culture” to “race” and “racism”. Multiculturalism is no longer just about ethnicity and culture, as it was conceived at the time of its adoption, and the Canadian identity the policy hopes to foster can no longer be framed within a settler-centric unity, as it was intended at the time of its adoption (Blanding 2021; Meister 2021). It has become evident that the Canadian identity of people from racialized communities is not just about cultural heritage, but depends more saliently on people’s racial identity, as it is socially constructed in Canada. A multiculturalism policy that evades race and racism can no longer be relevant in the current age of reconciliation and anti-racism, as demanded by racialized communities. A multiculturalism policy that trivializes race and racism can no longer be effective in the current times when all forms of racism, including overt and blunt type of macro-racism 1.0, institutional and systemic mezzo-racism 2.0, and micro-aggression type of racism 3.0, converge and co-exist (Fleras 2018). Although the Canadian Multiculturalism Act (1985) and other legislative efforts in the 1980s and 1990s added a component of equity and anti-racism, they may not be enough to encourage addressing racism in practice. As Reitz (2021) argued, although multiculturalism acknowledges problems facing racialized communities, it has not provided useful political resource or emphasized any obligations to address discrimination and disadvantage in action. This limitation contributes to multiculturalism being more of a rhetoric and to Canadians’ overall complacency with the current race relations in Canada.

## Beyond multiculturalism: toward pandemic anti-racism education

To look toward the future of a more equitable and just society of Canada that multiculturalism promises to achieve, it is high time that we go beyond multiculturalism with an action-oriented anti-racism education approach, which aims to address racism at macro, mezzo, and micro levels as racism operates through multi-level and intersected dynamics. Jean Augustine (2021), a former Member of Parliament and social advocate, states:

The challenges to inclusion…will not be met by having more cultural awareness events or by more studies on the experiences of racism…. Multiculturalism’s path forward for the next 50 years must be paved with the use of anti-racism and anti-oppression approaches shaping our collective understanding of the challenges to social inclusion. (p. 69)

In revisioning an alternative approach, we propose a pandemic anti-racism education model, which aims to call out any form of racism and xenophobia that is directly related to the global pandemic and eliminate racial oppression for achieving racial justice in post-COVID Canada (Guo and Guo 2021). Theoretically, this framework, as an alternative to the multiculturalism framework, is informed by critical race theory (CRT) that is deeply rooted in critique of racism and advocacy toward emancipation and social justice for racialized communities. It places the social construction of race as the central factor in affecting people’s life chances and ordering their social positions. It recognizes that racism is endemic and ordinary, pervasive in all aspects of society. CRT centres its analysis on power and power differentials, in particular, those engendered by racism, as racism constitutes the condition of an unequal society where the White sustains power and privilege through the oppression and marginalization of racialized communities (Luke 2009). CRT also recognizes the intersectionality of race, class, and gender in shaping people’s experience of social inequality, thus exposing multiple systems of subordination and oppression (Crenshaw 1991; Gillborn 2015). Emphasizing the historical, social, and economic context of racism, its key tenet is the recognition of race as a social and political construct (Matsuda et al. 1993). By being race-conscious, CRT offers an enabling framework to name and eliminate racial subjugation.

The application of CRT in the field of education has allowed for an understanding of social inequality in various educational settings from a race perspective. It has been an effective analytic tool for discussing issues of structural and institutional barriers to access power, knowledge legitimacy and democracy, and the language and cultural rights of racial minority groups (Howard and Navarro 2016; Powers 2007; Roithmayr 2000). At the same time, it raises the voices, experiences, and perspectives of racialized people (Ladson-Billings and Tate 1995). Although particularly focused on anti-Black racism when it enters the field of education, CRT is not a theorization of blackness, but rather, a theory of race and racism that applies to all oppressed racial groups (Delgado and Stefancic 2007; Dumas and Ross 2016). CRT promises to offer an alternative perspective to the multicultural discourse as experiences of discrimination, exclusion, and oppression are often not directly addressed in the multicultural discourses of inclusion, diversity, and equity (Lopez 2020). It functions as a critique of White supremacy and the limits of a hegemonic liberal form of multiculturalism (McLaren 1995; Melamed 2011). CRT can thus advance innovations in educational policy, research, and practice that are committed to a social justice agenda (Tate 1997).

Following CRT’s tenets, a pandemic anti-racism education model integrates multiple centres of knowledge; recognizes and respects for difference; affects social and educational change related to equity, access and social justice; and teaches for community empowerment (Dei 1996; Dei et al. 2001). In implementing this approach into education and social practices, the role of teachers and instructors should extend from the sphere of the classroom into the community and educational practices should engage with social and political issues. Educators need to explicitly teach pandemic anti-racism and develop awareness of discursive racialization and xenophobic violence and discrimination in relation to COVID-19 and discuss action plans to eliminate them. This requires collaboration among teachers, students, administrators, and community activists to work for change at a broader level.

A CRT-informed pandemic anti-racism education goes beyond the current multicultural model in three significant ways to effect social change. First, a pandemic anti-racism education shifts the policy dimension of multiculturalism to a focus on social justice. It means that cultural and socio-economic policies should consider issues of equality and equity for racialized communities. Inclusive policies should follow the three principles of social justice theorized by Nancy Fraser, that is recognition, redistribution, and representation (Fraser et al. 2004). Under these principles, anti-racism education model must recognize the cultures of racialized communities, ensure egalitarian redistribution of socio-economic resources, and problematize governance structures and decision-making procedures by increasing the visibility of racialized communities as decision-makers and representatives in consultation. Second, an empowering pandemic anti-racism education approach challenges the practice dimension of multiculturalism. It supports racialized communities to take initiatives in designing and implementing multicultural education programs that are not mere tokenistic celebrations of specific events, ethnic songs, dance, or rituals. Racialized individuals should be empowered to exercise their agency through funding support, awareness-raising education programs, and leadership programs focused on community development. Particularly, an anti-racist education model will not only engage students in articulating and reflecting on race and racism, but it will also challenge the “white gaze” (Rabelo et al. 2020), or knowledge premised on white supremacy, by encouraging the inclusion of multiple ways of knowing (Dei 1996; Guo and Guo 2021). Finally, a pandemic anti-racism education reflects a fundamental change in ideological focus. It means that education should confront ethnic relations and diversity in Canada as a politically mediated issue rather than as a matter confined to the domain of culture. An anti-racist model of education recognizes that systemic racism is still prevalent. Macro-level racism is not something in the past or something elsewhere. Racism does not just exist as isolated incidents or interpersonal trifles. Instead, an anti-racist model of education questions and decentres an exclusionary ideology of white supremacy in the social system and in political and public discourses. Multiculturalism that does not engage with anti-racism can at best achieve tolerance of cultural diversity and at worst, reproduces a Eurocentric racial hierarchy and white domination (Dei 2011).

As Kymlicka (2021) commented, Canada’s multiculturalism at present is deeply anchored in the chains of the past and at the same time looking forward with aspirations of rebirth in the future. The current conjuncture that situates multiculturalism’s 50th anniversary within a global pandemic clearly reflects how multiculturalism, with an inherent obscurity in meaning, is torn between an ideal of inclusion and equity, on the one hand, and a mere political rhetoric and unjust social reality, on the other. This paper re-visits Canada’s multiculturalism policy by reviewing the history and ethos of the multiculturalism policy and critically examining the current challenges to multiculturalism as they are manifested during Covid-19. With a focus on anti-Asian racism, anti-Black racism, and anti-Indigenous racism, this article reveals that racism is not something in the past in Canada. Rather, it is perceived and experienced by racialized communities at the present time during the pandemic. Experiences of racism indicate that multiculturalism has failed to address issues of inclusion and social justice as it has not challenged the fundamental social system of white supremacy, has not provided effective resources for confronting racism, and has not framed ethnic tensions as political. Considering these limitations, we hope the proposed pandemic anti-racism education sheds new light on the ongoing debate about multiculturalism that takes us beyond the traditional multicultural approach in building a more inclusive and socially just society in poast-Covid-19 Canada.

## Data Availability

Not applicable

## Footnotes

1 The term “Indigenous people” refers to the three groups of original inhabitants of the land that is now Canada as well as their descendants. As recognized by the Canadian Constitution, Indigenous people include First Nations, Inuit and Métis people. https://www.rcaanc-cirnac.gc.ca/eng/1100100013785/1529102490303
